# High-Throughput Quantitative RT-PCR in Single and Bulk *C. elegans* Samples Using Nanofluidic Technology

**DOI:** 10.3791/61132

**Published:** 2020-05-28

**Authors:** Laetitia Chauve, Jérémie Le Pen, Francesca Hodge, Pia Todtenhaupt, Laura Biggins, Eric A. Miska, Simon Andrews, Olivia Casanueva

**Affiliations:** 1 Babraham Institute; 2 Gurdon Institute, University of Cambridge; 3 Department of Genetics, University of Cambridge; 4 Wellcome Trust Genome Campus, Wellcome Trust Sanger Institute; 5 Laboratory of Virology and Infectious Disease, The Rockefeller University

**Keywords:** Genetics, Issue 159, RT-qPCR, single-worm, *C. elegans*, bulk samples, worm-to-CT, nematodes, high-throughput, nanofluidic, multiplex

## Abstract

This paper presents a high-throughput reverse transcription quantitative PCR (RT-qPCR) assay for *Caenorhabditis elegans* that is fast, robust, and highly sensitive. This protocol obtains precise measurements of gene expression from single worms or from bulk samples. The protocol presented here provides a novel adaptation of existing methods for complementary DNA (cDNA) preparation coupled to a nanofluidic RT-qPCR platform. The first part of this protocol, named ‘Worm-to-CT’, allows cDNA production directly from nematodes without the need for prior mRNA isolation. It increases experimental throughput by allowing the preparation of cDNA from 96 worms in 3.5 h. The second part of the protocol uses existing nanofluidic technology to run high-throughput RT-qPCR on the cDNA. This paper evaluates two different nanofluidic chips: the first runs 96 samples and 96 targets, resulting in 9,216 reactions in approximately 1.5 days of benchwork. The second chip type consists of six 12 x 12 arrays, resulting in 864 reactions. Here, the Worm-to-CT method is demonstrated by quantifying mRNA levels of genes encoding heat shock proteins from single worms and from bulk samples. Provided is an extensive list of primers designed to amplify processed RNA for the majority of coding genes within the *C. elegans* genome.

## Introduction

The optimization of single-cell RNA sequencing and qPCR revealed that transcriptional pulses or bursts can lead to massive variation in the number of RNA molecules per cell^1^. Further, these technologies uncovered substantial cellular heterogeneity previously missed by standard bulk transcriptomic measurements. Depending on the context, some single-cell transcriptional variability is caused by mixed cellular composition of tissues. However, even in isogenic cell populations grown under the same environment there is widespread transcriptional heterogeneity^2,3^. This ‘biological variability’ is increasingly identified as a ubiquitous property of cellular networks, from bacteria to man. In some cases, it can have phenotypic consequences in development, cancer progression, HIV latency, and response to chemotherapy^4,5^.

The nematode *Caenorhabditis elegans* is a unique model organism with ideal characteristics for studying the causes and consequences of biological variability between individuals. These nematodes are a simple model organism composed of 959 cells, and their transparent cuticle makes them amenable for in vivo imaging studies^6^. *C. elegans* is a hermaphroditic species that predominantly reproduces through self-fertilization; this resulted in isogenic laboratory strains. Despite isogenicity and controlled culture conditions, many phenotypes and transcripts are variable across individuals, suggesting that stochastic or microenvironmental differences contribute to heterogeneity across individuals^7,8^. Such variability in gene expression has multiple fitness consequences, including variability in the penetrance of mutations, survival, developmental timing, and fecundity^7,8,9^. Due to these features, single-worm studies provide the unprecedented opportunity to study biological variability in a whole organism.

There is a fundamental need in the field to develop and optimize technologies for accurate detection of transcripts at a single-worm level. New technologies, such as single-worm RNA sequencing^10^, RNA sequencing from isolated tissues^11^, and single-cell sequencing^12^ are now available for *C. elegans*. However, a main challenge remains: when monitoring interindividuality, weakly expressed genes often fall below detectable levels^13^. This is particularly relevant for rare transcripts isolated from small amounts of starting material, as there is a well-established, inverse relationship between mean expression and technical variance, often causing rare transcripts to fall below statistical cutoffs^13^. The optimization of high-throughput multiplexed qPCR technologies has proven useful for mammalian single-cell studies, in particular when studying the expression of rare transcripts^14,15^. This technology can also be used for benchmarking and validation purposes of other single-worm technologies.

**Figure 1:**
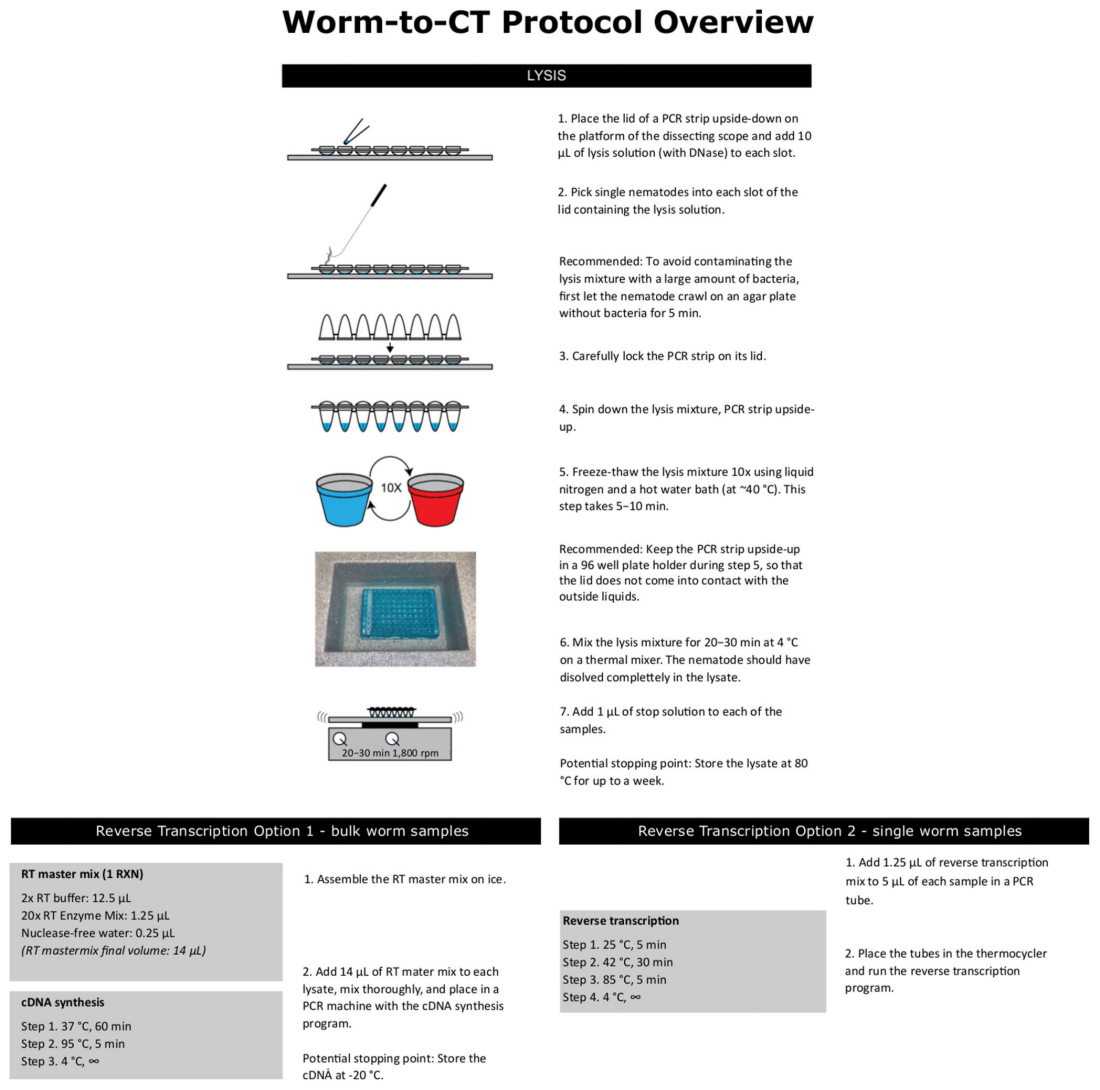
Overview of the Worm-to-CT Protocol. This figure shows a brief overview of the different steps required to run worms through the Worm-to-CT protocol. Two optional methods are shown for the reverse transcription step; these are interchangeable methods for either type of chip.

**Figure 2:**
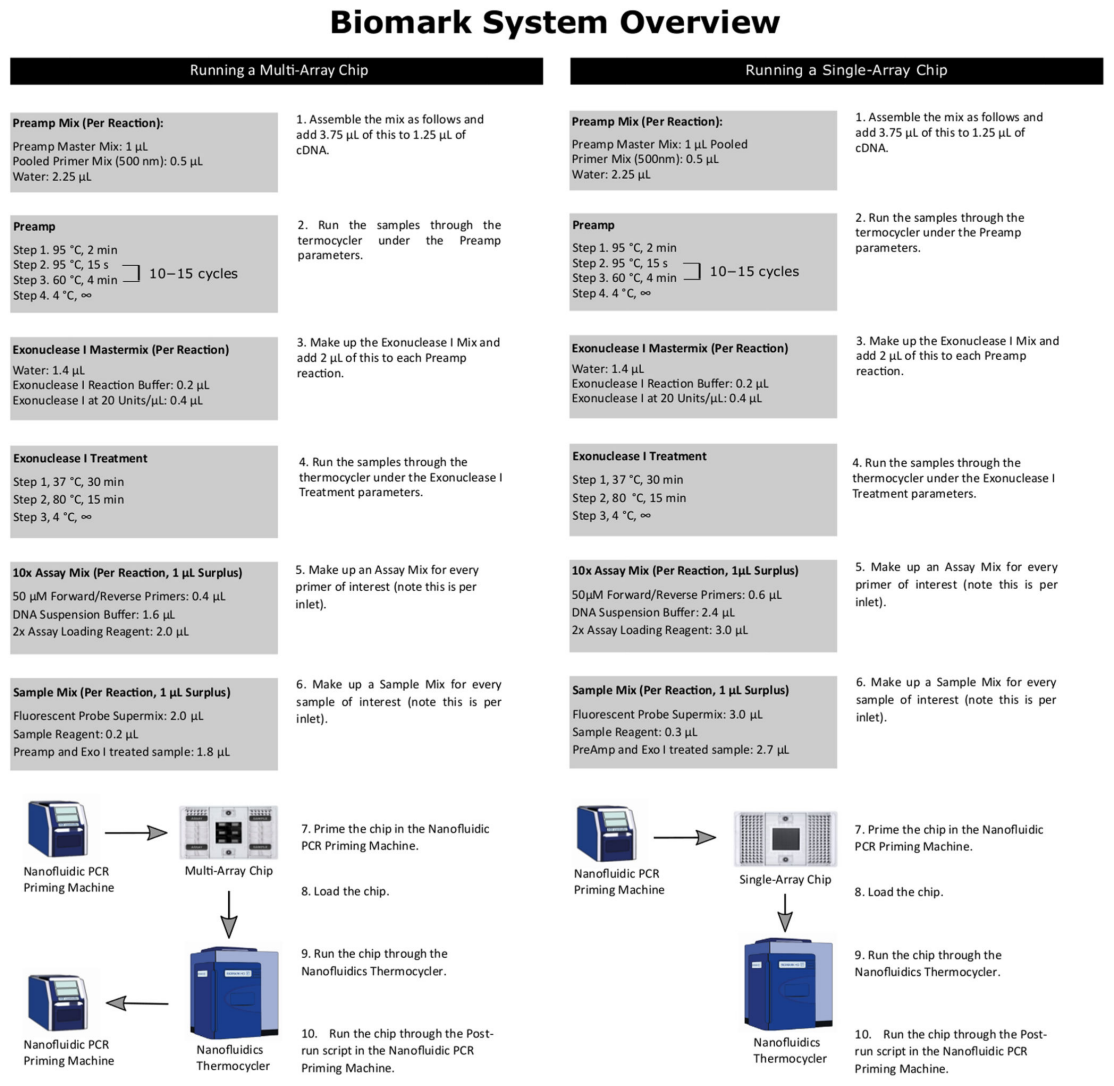
Overview of the preparation and running of nanofluidic qPCR. This figure depicts preparations for running the nanofluidic qPCR system using a multi-array chip and a single-array chip.

Worm-to-CT is a fast, robust method adapted from a kit used in cell biology studies, for single-worm cDNA preparation. cDNA obtained by this method coupled with multiplexed nanofluidic qPCR technology was chosen because it provides higher experimental throughput, a broader dynamic range of detection and has been validated for single-cell purposes^14,15^. The cDNA preparation described is also applicable for use with standard PCR technologies. The throughput is increased in two ways: First, cDNA preparation is faster and more reliable than traditional guanidium thiocyanate-phenol-chloroform extraction, because worms are directly added to the lysis buffer, skipping direct isolation of easily degradable RNA. Second, utilizing nanofluidic technologies significantly increases the number of samples and targets that can be run simultaneously. In this paper, two chips are compared: a single-array chip and a multi-array chip. A single-array chip can run 96 single worms and 96 primer sets, resulting in 9,216 reactions per experiment. To accomplish a similar throughput using standard qPCR technologies would require 96 separate qPCR experiments, using 96 well plates. The smaller and more flexible multi-array chip consists of six 12 x 12 arrays resulting in 864 reactions. The method’s superior reliability and sensitivity are boosted by nanofluidic technology and by the introduction of a preamplification step. The method presented in this paper is meant to be used together with a state-of-the art statistical algorithm to extract biological variance. This article presents the protocol for rapid cDNA preparation and high-throughput qPCR for both single-worm and batch worm samples; the algorithm will be published elsewhere. For this protocol, the organization of each chip should be prepared prior to the experiment. Table 1 and Table 2 show examples of these plans for a multi-array and single-array chip, respectively. There are also overviews of the Worm-to-CT protocol detailed in Figure 1 and running the multi-array and single-array chips in Figure 2.

## Protocol

NOTE: Throughout this protocol *Caenorhabditis elegans* is referred to as “worm” or “worms”. A variety of *C. elegans* strains can be ordered through online databases or by directly contacting labs that use the model organism. Part I of this protocol (sections 1–3) describes cDNA preparation through the Worm-to-CT protocol. Part II of this protocol (sections 4-13) describes running high-throughput RT-qPCR using nanofluidics, adapted from a protocol developed by Fluidigm^16^. This protocol applies to the use of the two types of nanofluidic chips defined earlier, the single-array chip, which can monitor 96 targets into 96 samples (9,216 RT-qPCR reactions total), or the multi-array chip, which functions as subunits of 12 target x 12 samples. Every multi-array chip contains six independent arrays that can be run together or separately. For instance, using a whole multi-array chip can monitor 72 targets x 12 samples (or vice versa), or 36 targets x 24 samples (or vice versa). For further information regarding any of the materials used in this protocol, refer to the **Table of Materials**.

### 1 RT-qPCR primer validation

NOTE: Real-time primers were designed based on the recommended properties originally issued by MIQUE guidelines^17^. To make primers specific for processed RNA, products were designed such that the two primers bound to either side of at least one splice junction. Requirements for suitable primers included 20%-80% guanine and cytosine content, a melting temperature of 58-60 °C, a difference in melting temperature between primer pairs of ≤0.5 °C and a product length of 70–120 bp. The sequence of the primers generated can be found in Supplemental Table 1. Open source code for the scripts used to generate the primers can be found at https://github.com/s-andrews/wormrtpcr. Primer pairs for transcripts with splice sites were designed so that they lie in two exons flanking an intron but were not designed to be splice-variant specific, using NCBI Primer Blast software^18^. For this study, the primer sets were blasted against the *C. elegans* genome to test for any off-target complementarity.

1. Retrieve primers from the database of RT-qPCR primers (Supplemental Table 1). Alternatively, design qPCR primer pairs using online tools such as NCBI Primer Blast^18^.
2. Perform a qPCR standard curve to monitor specificity and PCR efficiency for each pair of primers using standard bulk qPCR techniques^19^ and following MIQUE guidelines^17,20^.
  NOTE: Only primer pairs with R^2^ > 0.98 and PCR efficiency between 85% and 115% should be used. The sequence, PCR efficiency, and R^2^ for the primers used in this study are detailed in Table 3.

### 2 Worm lysis through Worm-to-CT

1. Pick the worms from their bacterial lawn onto a fresh, unseeded NGM plate and allow the worms to move around the plate for 5 min to remove most of the bacteria from the worm through its movement.
  NOTE: The bacterial lawn and growing conditions will differ depending on the experimental design. The experiments presented here require 6 cm NGM plates seeded with OP50 *Escherichia coli* with worms of interest grown to stage L4.9 in a 20 °C incubator.
2. In an RNase-free hood, prepare a master mix consisting of 12.5 μL of 2x RT buffer, 1.25 μL of 20x RT enzyme buffer, and 0.25 μL of nuclease-free water per sample. Add 14 μL of master mix to 11 μL of each sample from step 2.8.
3. Place the lid of a PCR strip upside down on the platform of the dissecting scope and add 10 μL of the lysis mix to domed PCR tube caps under a compound microscope.
  NOTE: With only one or two samples, it is better to use a PCR strip containing at least four tubes, as this reduces the risk of the caps blasting open in the subsequent freeze-thaw steps. Alternatively, rubber bands can be used to hold the caps in place. In that case ensure caps are properly closed every time the tubes are transferred.
4. Pick the worms from the plate into each slot of the lid containing the lysis mix by “scooping” them (i.e., catching the worms underneath with the pick) to avoid bacterial contamination. Close the tubes and spin them down for 5 s using a tabletop microcentrifuge (**Table of Materials**) before placing them in a Dewar flask filled with liquid nitrogen.
  NOTE: Between 15 and 30 worms should be used for bulk experiments and 1 worm for single-worm measurements.
  CAUTION: When handling liquid nitrogen wear Cryo-Gloves as well as protective eyewear and adhere to standard clothing regulations, because contact with the skin or eyes can cause serious frostbite injury.
5. Freeze-thaw the PCR tubes 10x by transferring them between liquid nitrogen and a ~40 °C water bath. Leave the tubes in the liquid nitrogen for a minimum of 5 s to ensure the samples are completely frozen. Leave the tubes in the water bath until the samples thaw. Do not leave in for a longer period, as this leads to RNA degradation.
  NOTE: Tubes can be left in liquid nitrogen for an extended period of time, as samples are frozen to approximately -200 °C, reducing RNA degradation. This should not, however, be a protocol stopping point, because liquid nitrogen evaporates rapidly.
6. Mix the samples on a thermal mixer (**Table of Materials**) set at 4 °C for 20-30 min rotating at ~1,800 rpm.
7. While the samples are being mixed, thaw the stop solution on ice.
8. Spin the samples down using a tabletop microcentrifuge (**Table of Materials**) and add 1 μL of stop solution to each tube.
  NOTE: The samples can be left at -80 °C for up to 1 week before reverse transcribing the RNA (section 3).

### 3 Reverse transcription

NOTE: For reverse transcription of single worms, the results shown here were generated using the reagents provided with the nanofluidic chips (option 2 in Figure 1). The reagents highlighted in option 2 of Figure 1 were also used for reverse transcription of pooled samples. Either method works interchangeably for the different sample types.

1. Reverse transcription of single worms
  1. In an RNase-free hood, add 1.25 μL of reverse transcription mix (**Table of Materials**) to a fresh PCR tube.
    NOTE: A 96 well PCR plate and an automatic pipette can be used if there are many samples. The manufacturer’s protocol states that 1 μL can used per sample.
  2. Take 5 μL of the lysis solution and stop solution mix from step 2.8 and add it to the fresh PCR tube containing the reverse transcription mix.
    NOTE: The manufacturer’s protocol states that 1 μL of RNA (2.5 pg/μL–250 ng/μL) can be used per reaction. A negative RT control per plate can be added by replacing the reverse transcription mix with 5 μL of lysed sample and 1.25 μL of RNAse-free water.
  3. Run the samples using the following reverse transcription program on a thermocycler: 25 °C for 5 min, 42 °C for 30 min, 85 °C for 5 min and 4 °C for ^∞^.
    NOTE: The cDNA produced can be stored at -20 °C before proceeding to amplification and data collection using high-throughput qPCR.

- Reverse transcription for bulk samples (15–30 worms)
  1. In an RNase-free hood, prepare a master mix consisting of 12.5 μL of 2x RT buffer, 1.25 μL of 20x RT enzyme buffer, and 0.25 μL of nuclease-free water per sample. Add 14 μL of master mix to 11 μL of lysis solution and stop solution mix from step 2.8.
    NOTE: When dealing with a large number of samples this can be performed in 96 well plates.
  2. Run the samples through a thermocycler using the following reverse transcription program: 37 °C for 60 min, 95 °C for 5 min, and 4 °C for ^∞^.
  3. Dilute the produced cDNA 1:4 in nuclease-free water.
    NOTE: Generally, the products come to a final volume of 25 μL, in which case 75 μL should be added. However, due to condensation the final volume can vary. Therefore, adjust accordingly to make a 1:4 ratio in the final solution. This dilution step does not apply if performing qPCR on single worms. The cDNA produced can be stored at -20 °C before proceeding to amplification and data collection using high-throughput qPCR.

### 4 Preparing the multiplex primer mix

1. Prepare a forward/reverse (F/R) primer stock for each pair of primers at 50 μM final concentration. Mix the same volume of forward and reverse primers at 100 μM each.
2. Combine 1 μL of 50 μM F/R primer stock for each primer pair to be tested. Add the DNA suspension buffer up to a total volume of 100 μL.
  NOTE: The stock primer concentrations here differ from those described in the manufacturer’s protocol^16^ but retain the same final concentration of 500 nM.

### 5 Target specific preamplification

1. Prepare a master mix containing 1 μL of preamplification mastermix (**Table of Materials**), 0.5 μL of the pooled primer mix (step 4.2), and 2.25 μL of nuclease-free water per reaction with a 10% overall surplus volume.
2. In a 96 well plate, aliquot 3.75 μL of the master mix into as many wells as required for the number of samples to be run.
3. Add 1.25 μL of the cDNA solutions of interest generated at step 3.1.3 or 3.2.3 to each well.
4. Cover the plate with 96 well sealing tape, briefly vortex, and centrifuge with a tabletop plate spinner. Transfer to a thermocycler and run the following program: 95 °C for 2 min, 15 cycles of denaturation at 95 °C for 15 s, annealing/extension at 60 °C for 4 min, and 4 °C for ^∞^.
  NOTE: The manufacturer recommends from 10–20 cycles for the preamplification reaction^16^. This protocol recommends 10 or 15 cycles depending on the expression levels of the target genes.

### 6 Exonuclease I treatment

NOTE: This is to remove unincorporated primers from preamplification.

1. Prepare an exonuclease I mix containing 0.2 μL of exonuclease I reaction buffer (**Table of Materials**), 0.4 μL of exonuclease I at 20 U/μL (**Table of Materials**), and 1.4 μL of nuclease-free water per sample. Keep all reagents on ice, especially the exonuclease I.
2. Remove the 96 well plate (step 5.4) from the thermocycler, centrifuge with the tabletop plate spinner, and carefully remove the seal. Add 2 μL of the exonuclease I mix to each preamplification reaction. Reseal, centrifuge, and place the 96 well plate back into the thermocycler using the following program: 37 °C for 30 min, 80 °C for 15 min, and 4 °C for ^∞^.
3. Take the samples out of the thermocycler and dilute them 1:5 by adding 18 μL of 1x Tris EDTA buffer (**Table of Materials**).
  NOTE: It is possible to keep the cDNA samples at -20 °C for later use. The manufacturer’s protocol suggests potential dilutions of 5x, 10x, or 20x at this stage^16^, depending on the expression level of the targets of interest.

### 7 Preparing the assay mixes

NOTE: Assay mixes can be prepared in 384 well plates, as the wells have the same spacing as the nanofluidic chips, making loading easier.

1. Preparing assay mixes for a multi-array chip
  1. Prepare a master mix consisting of 2 μL of 2x assay loading reagent (**Table of Materials**) and 1.6 μL of DNA suspension buffer (**Table of Materials**) for each well according to the prepared plan. Aliquot 3.6 μL of this master mix per well into a 384 well plate.
  2. Add 0.4 μL of the 50 μM F/R primer stock prepared in step 4.1 to the appropriate wells according to the prepared plan.
    NOTE: This provides a total of 4 μL of assay mix per well, with a surplus of 1 μL.
2. Preparing assay mixes for a single-array chip
  1. Prepare a master mix consisting of 3 μL of 2x assay loading reagent and 2.4 μL of DNA suspension buffer for each well according to the prepared plan. Aliquot 5.4 μL of this master mix per well into a marked 384 well plate.
  2. Add 0.6 μL of the 50 μM F/R primer stock to the appropriate wells according to the prepared plan.
    NOTE: This provides a total of 6 μL of assay mix per well, with a surplus of 1 μL.

### 8 Preparing the sample mixes

NOTE: Sample mixes can be prepared up to 1 day in advance and stored at 4 °C.

1. Preparing samples for a multi-array chip
  1. Prepare a sample master mix consisting of 2 μL of 2x fluorescent probe supermix with low ROX (**Table of Materials**) and 0.2 μL of sample reagent (**Table of Materials**) per sample. Dispense 2.2 μL of this mix into the marked 384 well plate.
    NOTE: The manufacturer recommends not vortexing the sample reagent^16^.
  2. Pipette 1.8 μL of each preamplified, exonuclease I treated sample from step 3.2.3 into the appropriate wells according to the prepared plan.
    NOTE: This gives a total of 4 μL, with a surplus of 1 μL.
2. Preparing samples for a single-array chip
  1. Prepare a sample master mix consisting of 3 μL of 2x fluorescent probe supermix with low ROX (**Table of Materials**) and 0.3 μL of 20x DNA-binding dye sample loading reagent (**Table of Materials**) per sample. Dispense 3.3 μL of this mix into the marked 384 well plate.
  2. Pipette 2.7 μL of each preamplified and exonuclease I treated sample from step 6.3 into the appropriate wells according to the prepared plan.
    NOTE: This gives a total of 6 μL, with a surplus of 1 μL. If there are any wells to be run without a sample these must be loaded with sample master mix and 2.7 μL of water instead of cDNA. This is recommended for both chip types. The machine needs low ROX in every inlet in order to detect the chip’s grid.

### 9 Priming the nanofluidic chip

NOTE: A multi-array chip only needs to be primed on the first run. If there are subsequent runs of the same chip this stage can be skipped. These steps are the same for both chip types.

1. Slowly and carefully, inject the full 150 μL of the control line fluid from the syringes included into the accumulators of the chip. Ensure that no control liquid touches the chip by holding the chip at a 45° angle and holding the tip of the syringe away to avoid spillage.
2. Remove the blue protective film from the bottom of the chip.
3. Place the chip into the nanofluidics PCR priming machine (**Table of Materials**), with the barcode facing outwards. Run the ‘**Prime (153x)**’ script, which takes ~15–20 min.
  NOTE: Turn on the nanofluidics thermocycler (**Table of Materials**) at this stage, because the camera takes about 10 min to cool down to below 0 °C.

### 10 Loading the nanofluidic chip

1. Remove the barrier plugs sequentially as loading takes place. This reduces the chance of misloading wells.
2. Transfer either 3 μL for a multi-array chip or 5 μL for a single-array chip of each primer assay mixes and sample mixes to the corresponding inlets of the nanofluidic chip according to the prepared plan. Make sure not to introduce bubbles, which can cause the total volume transferred to be smaller than desired.
  NOTE: If there are any wells that will be run without a primer it is important for these to be loaded with master mix, substituting the primer volume with water. This applies to both chip types. At this stage, it can be easier to place the chip on a dark surface, which will allow the wells to be seen more easily.

### 11 Running the nanofluidic chip

NOTE: The first time running a multi-array chip, set up the tracking file by selecting **Tools** | **Flex Six Usage Tracking**, click **New**, enter a file name, and select a location before clicking **Done**.

1. Open the data collection software. Click **Start New Run**. Place the loaded chip into the nanofluidics thermocycler with the barcode facing outwards.
2. Choose the project setting if applicable, then click **Next** | **Load**. If loading a multi-array chip, select the partitions (arrays) to be run.
3. Select the application **Reference Probes**, then change the application type to **Gene Expression**, and change the passive reference to **ROX**. Select the **Single Probe Assay**, change the probe type to **Eva Green**, and click **Next**.
4. Select the thermal cycling protocol **GE FLEX six Fast PCR+Melt v1** to run a multi-array chip, or the protocol **GE 96.96 Fast PCR+Melt v2** to run a single-array chip.
5. Confirm that **Auto Exposure** is selected and click **Start Run**.

### 12 Post chip run

NOTE: This section is only necessary for multi-array chips when not using the entire chip.

1. Take the chip out of the nanofluidics thermocycler and load into the nanofluidics PCR priming machine and run the Post Run (153x) script, which lasts 5 min.
2. Label the plugs used for personal reference.
  NOTE: The chip can now be stored at room temperature and the remaining arrays on the chip can be run within 2 months.

### 13 Data cleanup and analysis

1. Open the data in the ‘**Real-Time PCR Analysis**’ software (**Table of Materials**). Check the melting peak temperature for every primer pair tested. Eliminate results exhibiting more than one melting temperature peak, for a given primer pair.
  NOTE: Multiple peaks only appear occasionally, presumably when primer pairs forms dimers, or from interactions of target primers with other primers in the pooled primer mix.
2. Export the data as a ‘heatmap’ spreadsheet file and eliminate failed samples or primers.
3. Analyze data using the standard Delta-Ct method^21^. For statistical evaluation perform one-way ANOVA on relative expression levels.

## Representative Results

### Validation of Worm-to-CT as a cDNA preparation method

**Figure 3:**
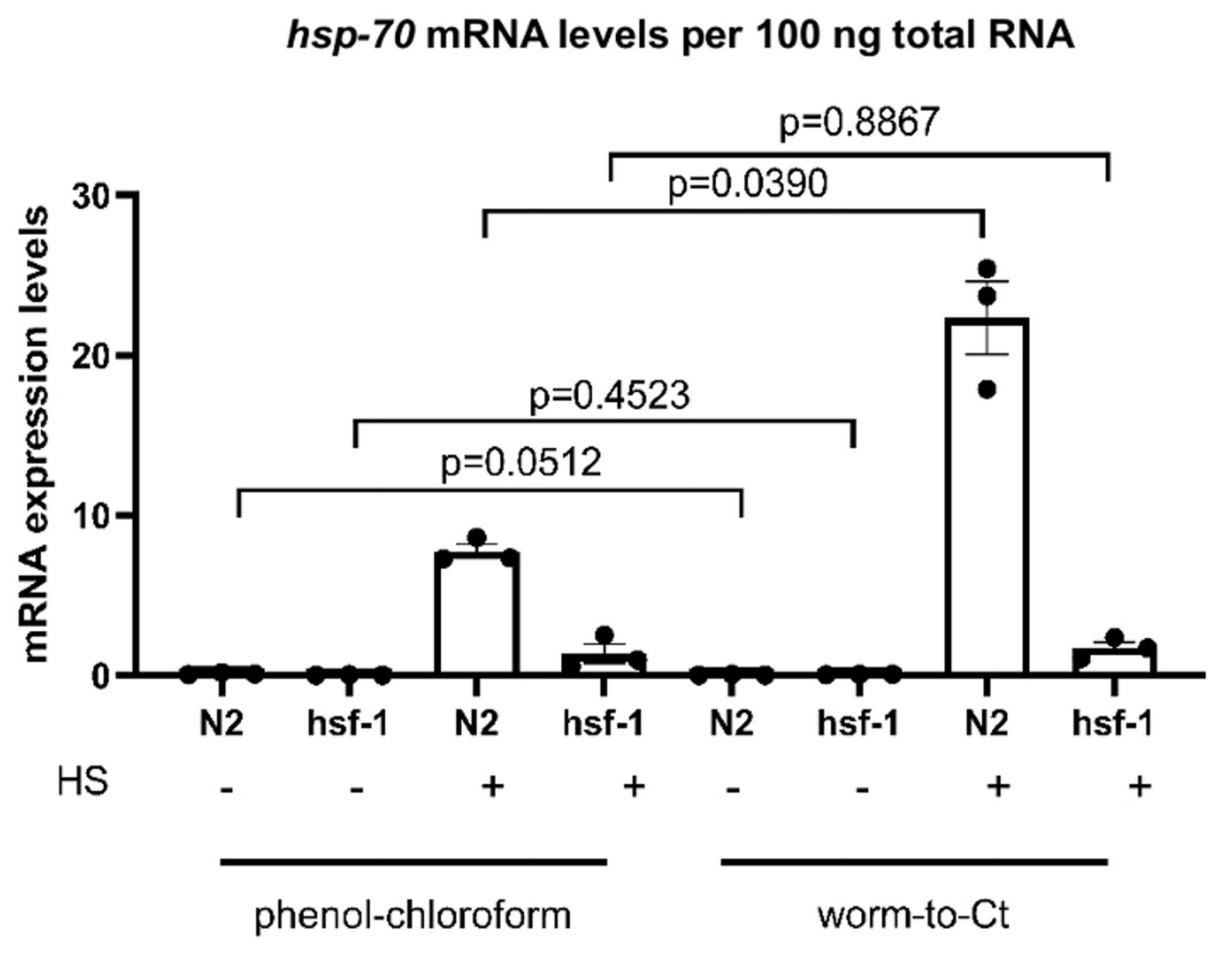
Worm-to-CT protocol on bulk samples provided reliable results. Comparison of Worm-to-CT protocol versus regular guanidium thiocyanate-phenol-chloroform extraction^22^ on bulk samples. Consistent with previous findings, in *hsf-1(sy441*) mutants^23^, the levels of *hsp* transcripts in response to heat shock decreased. The above histograms depict the induction of *hsp-70* in the absence of (-), or following (+) a short heat shock of 30 min at 34 °C. The cDNA was obtained using guanidium thiocyanate-phenol-chloroform extraction applied to 1,000 worms (left) or using the Worm-to-CT method applied to 30 pooled worms (right). The expression levels of *hsp-70* per 100 ng of total RNA obtained by each method were compared. As expected, in *hsf-1(sy441)* the transcriptional induction of *hsp-70* in response to heat shock significantly decreased by 82.7% using guanidium thiocyanate-phenol-chloroform and by 92.3% using the Worm-to-CT method. The mRNA levels from target genes were normalized against the average of the three housekeeping genes *cdc-42*, *pmp-3*, and *ire-1*. Each dot represents a biological replicate. Data were log transformed for statistical analysis, as they did not meet the conventions required for parametric analysis. Statistical analysis was done using a RM-One-way ANOVA using Sidak’s multiple comparisons test. Wild type = N2, hsf-1 = *hsf-1(sy441)*. Bars denote the standard error of the mean.

To test if the Worm-to-CT protocol is a valid cDNA extraction method, it was compared to standard guanidium thiocyanate-phenol-chloroform extraction methods. The results are shown in Figure 3, where cDNA was prepared from an average of ~1,000 worms using standard guanidium thiocyanate-phenol-chloroform extraction techniques^22^ and from 30 worms using the Worm-to-CT method. The samples were heat shocked simultaneously (30 min at 34 °C). Globally, *hsp-70* mRNA expression levels per 100 ng of total RNA were comparable using both methods. However, in the case of highest *hsp-70* expression (i.e., in N2 following heat shock) expression levels were higher with the Worm-to-CT method, indicating improved sensitivity.

To determine if an expected decrease in *hsp* expression in *hsf-1(sy441)*^23^, a mutation in the main transcriptional regulator of molecular chaperones^23,24^, could be reproduced, transcriptional chaperone induction following a brief heat shock was compared. With both methods a decrease in *hsp-70* induction was detected in *hsf-1(sy441)* animals. This was expected, because mutant *hsf-1(sy441)* animals exhibit a decreased ability to induce chaperones due to a truncation in the transactivation domain of HSF-1. For guanidium thiocyanate-phenol-chloroform extraction *hsp70* decreased by 82.7% compared to controls and 92.3% for Worm-to-CT compared to wild type animals (Figure 3). The results were comparable between both methods and comparable to previous reports^23^. These results indicate that the Worm-to-CT method is a valid alternative to standard cDNA synthesis techniques.

### Validation of the nanofluidics PCR platform used to amplify mRNA targets

**Figure 4:**
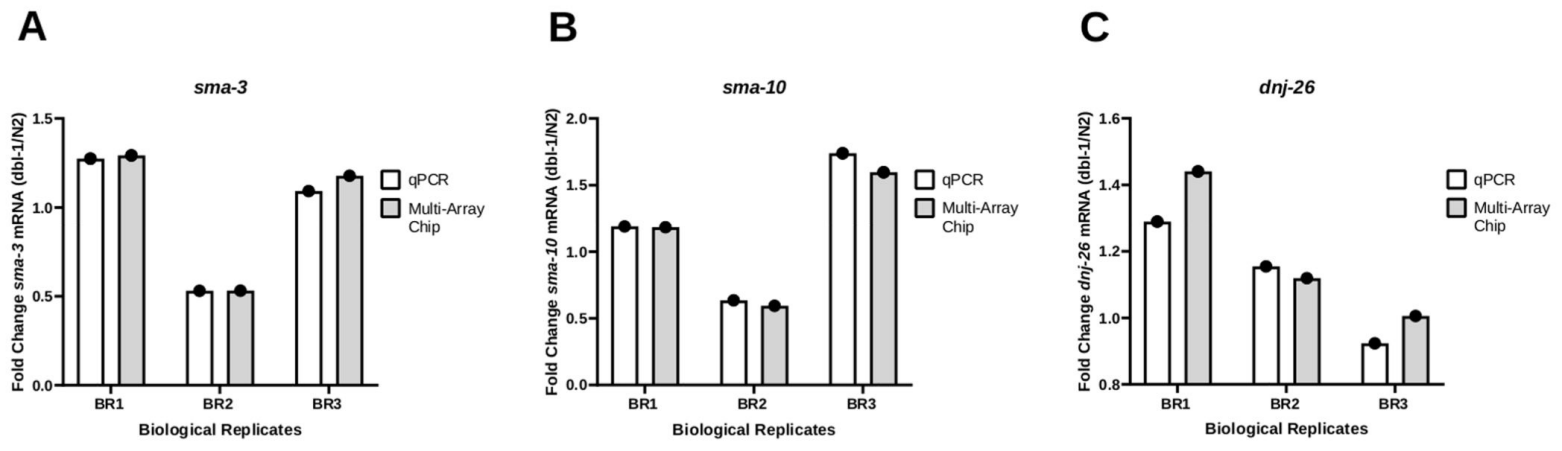
Expression patterns were consistent between standard qPCR and nanofluidic qPCR systems. (**A**) The expression level of *sma-3* (A), *sma-10* (B) or *dnj-26* (C) mRNA was determined through regular qPCR and nanofluidic qPCR (multi-array chip) from three biological replicates of cDNA generated through Worm-to CT from the wild type strain (N2) and the *dbl-1(nk3)* knockout strain^25^. Relative mRNA expression levels were determined for each strain using the Delta-Ct method^21^. Fold change was then determined by dividing the expression levels obtained in *dbl-1(nk3)* worms by the corresponding mRNA levels in the N2 strain. As shown in panel **A**, the patterns were consistent for both methods in each individual biological replicate. (**B**) and (**C**) are the same as (**A**) for *sma-10* and *dnj-26* mRNA levels, respectively. Target mRNA levels were normalized against the housekeeping genes *cdc-42* and *pmp-3*. Statistical analysis was calculated for each gene using a paired t-test comparing the results of the three biological replicates produced through standard qPCR and those generated through nanofluidic qPCR. The P-values of these comparisons were 0.3113, 0.2635, and 0.3481 for *sma-3*, *sma-10*, and *dnj-26*, respectively.

To test the consistency of the results using nanofluidic qPCR for transcript amplification, the PCR results obtained from the Worm-to-CT bulk method were compared on both a standard qPCR system (**Table of Materials**) and a nanofluidic qPCR system using a multi-array chip. The fold change in the expression of three different genes, *sma-3* (Figure 4A), *sma-10* (Figure 4B), and *dnj-26*, was monitored (Figure 4C) in animals carrying a null allele in *dbl-1* (*dbl-1(nk3)*)^25^ compared to wild type counterparts*. Dbl-1* encodes the sole ligand of the Bone Morphogenetic Protein (BMP) signaling pathway. *sma-3* and *sma-10* are genes encoding SMAD orthologues, key components of the BMP signaling cascade. *Dnj-26* encodes a molecular chaperone, a target of BMP signaling. These results show little to no difference in the fold change comparing the results of the two methods, resulting in not significant P-values at 0.3113, 0.2635, and 0.3481 for *sma-3*, *sma-10*, and *dnj-26*, respectively. Altogether, these results show that the Worm-to-CT method applied to bulk samples is an efficient and rapid way to extract RNA from few worms and provides reliable data when coupled with either standard PCR systems or high-throughput nanofluidics-based qPCR platforms.

### Comparison between the expression levels obtained by bulk samples with averages obtained from single worms

**Figure 5:**
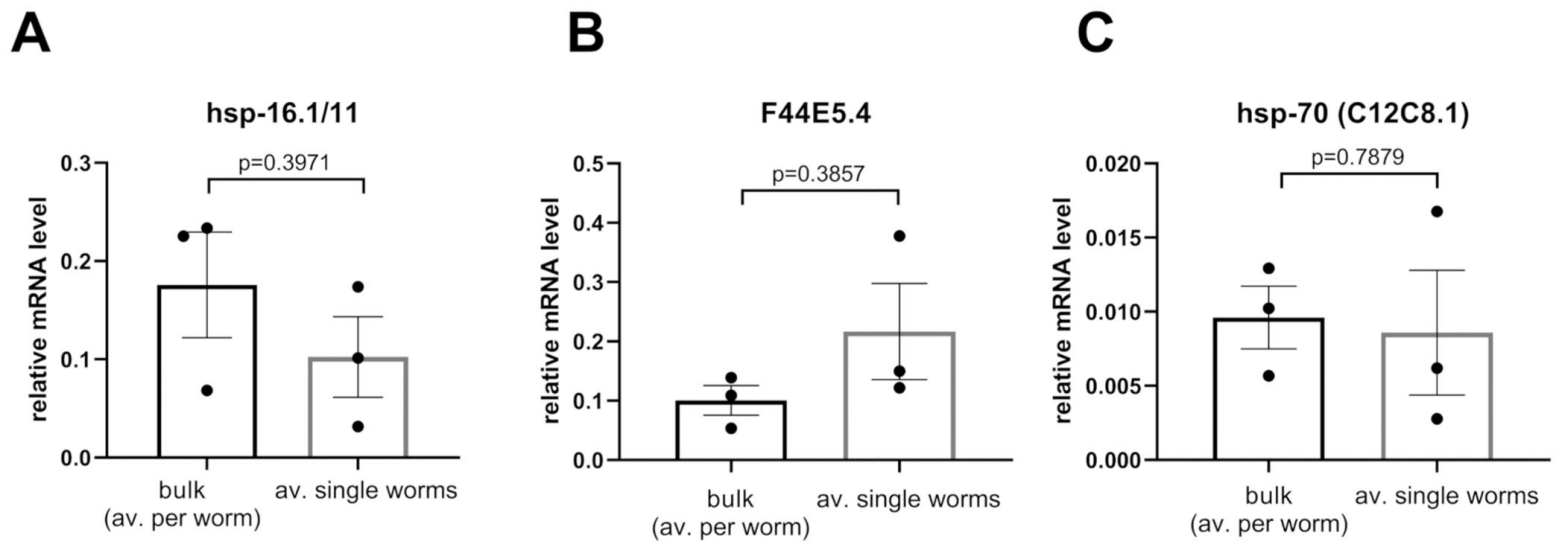
Using Worm-to-CT method on bulk samples or on single worms provided similar levels of expression when normalized per worm. The expression levels of (**A**) *hsp-16.1/11*, (**B**) F44E5.4, and (**C**) *hsp-70* (C12C8.1) were analyzed in young adult animals in the absence of heat shock either by performing Worm-to-CT on a bulk of 25 animals, or on 36 single individuals. When the data were normalized per worm, there was no significant difference between levels obtained per worm for each transcript using both methods. The mRNA levels from target genes were normalized against the average of the three housekeeping genes *cdc-42, pmp-3*, and *ire-1*. Bars represent the standard error of the mean. Statistics = paired t-test.

The relative expression levels were calculated using either cDNA obtained from bulk samples (25 worms) or from an average of 36 single worm samples (Figure 5). Both cDNAs were obtained using the Worm-to-CT method and amplified using nanofluidics PCR technology. As observed in Figure 5A–C, for all chaperones tested (i.e., *hsp16.1*, F44E5.4, *hsp-70*), the methods detected comparable expression levels. These results indicate that parameters obtained from single worms are reliable.

### Application of Worm-to-CT coupled to nanofluidics technology to estimate single-worm gene expression parameters

**Figure 6:**
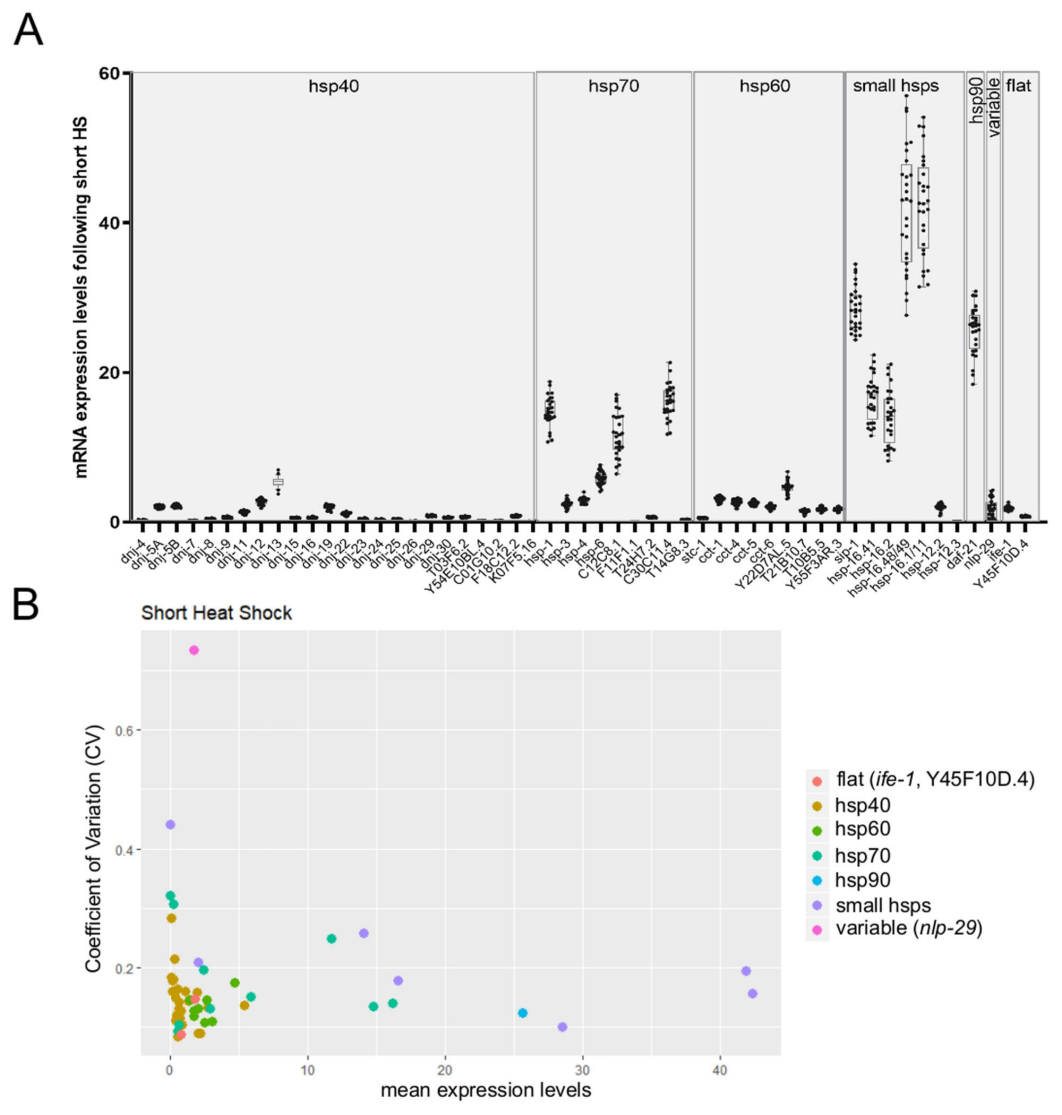
High-throughput RT-qPCR on single worms using the Worm-to-CT method could monitor inter-individual variability in gene expression. (**A**) The mean expression levels for 53 transcripts obtained upon exposure to a short heat shock (30 min at 34 °C). Boxplots represent the distribution of mean mRNA expression from individual worms (an average of three technical replicates were used per individual worm). The dots represent expression levels in 28 individual worms. The mRNA levels from target genes were normalized against the average of the three housekeeping genes *cdc-42, pmp-3*, and *ire-1*. (**B**) The coefficient of variation^26^ (CV) as a function of mean mRNA expression for 53 transcripts following exposure to a short heat shock was calculated from 28 individual animals (raw data shown in panel **B**). The set of transcripts includes the variable *nlp-29* transcript^27^ and two stable transcripts (*ife-1* and *Y45F10D.4*; unpublished data). The CV is the ratio of the standard deviation to the mean. This CV was utilized to estimate inter-individual variability in transcript expression between individual worms. As expected, inter-individual variability scaled with decreased mean expression levels.

Because the single-array chip allows monitoring of up to 96 target transcripts on 96 individual samples, it is therefore well-suited to monitor individual variability in transcript expression between single worms. Figure 6A presents a representative result showing the mean expression of multiple *hsp* transcripts from single worms following a short heat shock. As observed in the figure, the variability in the expression of transcripts differed dramatically across different genes (Figure 6A). To gain further insight, the coefficient of variation (CV) was calculated by dividing the standard deviation by the mean of the expression levels^26^ (Figure 6B). Three genes whose CV values have been previously estimated by alternative methods were monitored (unpublished data). Two stable transcripts (*ife-1* and *Y45F10D.4*) and one variable (*nlp-29*^27^) showed their expected variability. The graph also clearly depicts the well-known inverse relationship between variability values and expression levels^26^ (Figure 6B).

**Figure 7:**
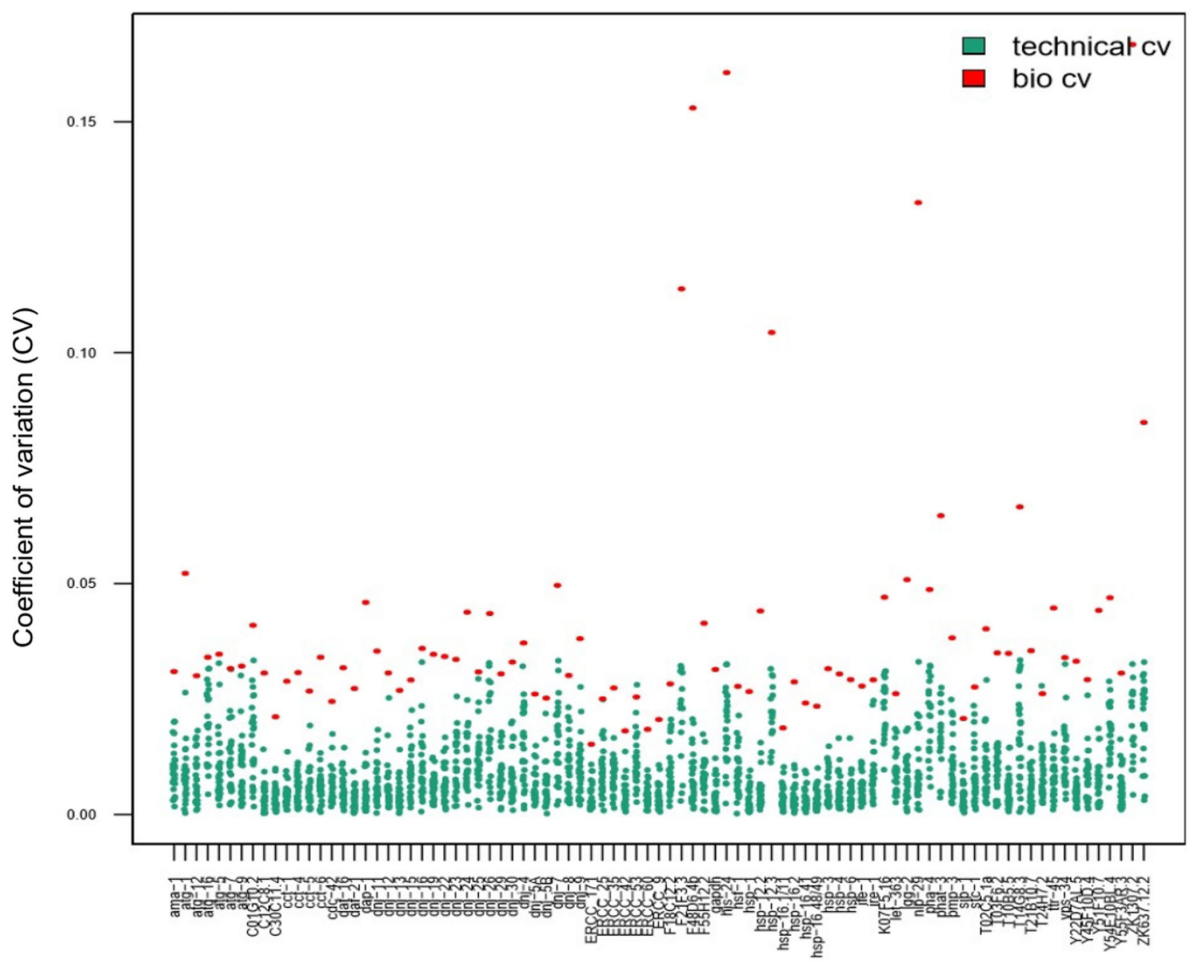
Technical replicates were not necessary when analyzing inter-individual variability in gene expression using a nanofluidic chip. The data presented in this graph were obtained in 28 individual worms following a short heat shock (30 min at 34 °C). Each red dot represents the coefficient of variation (CV) of mean transcript expression levels for one transcript assayed between 28 individual worms (bio CV). Each blue dot represents the CV of expression levels between three technical replicates obtained from a single worm, per transcript assayed (technical CV). This graph shows that technical variability (between technical replicates) was much lower than biological variability (between individual worms), suggesting that it is unnecessary to perform technical replicates on a nanofluidic gene expression array when assaying gene expression in single worms, similarly to single-cell studies^14,15,28^.

Technical replicates are of paramount importance to ensure reproducibility when using bulk samples. However, this is not necessarily the case for single-cell experiments^14,15,28^. To determine if the use of technical replicates is necessary for parameter estimation when using single-worm samples, 28 individual worms were harvested, following a short heat shock, and processed using technical triplicates. The CV values calculated from single-worm data obtained in triplicate (blue dots in Figure 7, technical CV) versus those for every transcript obtained from individual worms (red dots in Figure 7, biological variability) were compared. For every transcript tested, the technical CVs were lower than the biological CVs, indicating that technical triplicates were not required for parameter estimation. The fact that technical replicates are not required increases the throughput of the experiment without compromising quality.

## Discussion

In this paper, the Worm-to-CT protocol is shown to be a rapid and efficient method to extract RNA from single worms or a small pool of worms. The high-throughput offered by the nanofluidic system makes it ideal for quantification of inter-individual variability measurements. Furthermore, the high sensitivity of this method allows the detection of genes expressed at low levels that fall below detection when using single-worm RNA-seq technologies^9^.

When considering the choice of method to prepare cDNA from single worms. Ly et al.^29^ optimized a protocol that relies on proteinase K for cuticle digestion. The cuticle is a major hurdle for the isolation of molecules from worms and proteinase K provides an effective method to break it. However, proteinase K has to be heat-inactivated to be able to use enzymes for reverse transcription. While Ly et al. used a 10 min exposure to 96 °C, this step was avoided in this protocol because RNA is easily degradable. Instead of using proteinase K, repeated freeze-thaw cycles were used to break the cuticle. The freeze-thaw is an effective method to break the cuticle because more RNA can be isolated per worm. Ly et al. report that the total RNA extracted per worm is 35 ng using proteinase K, whereas this protocol obtains 51.75 ng ± 6.74 SEM of total RNA per worm. Avoidance of heat exposure coupled with preamplification steps apparently widens Worm-to-CT’s dynamic range of detection compared to standard protocols. Ly et al. report absolute Ct values of 21.1 ± 0.15 for *hsp-16.2* and 22.8 ± 0.17 for *hsp-70* after heat shock. Using the same heat shock conditions (1 h at 30 °C), this protocol obtains absolute Ct values of 17.93 ± 0.57 for *hsp-16.2* and 21.13 ±0.33 for *hsp-70*. This indicates that the freeze-thaw lysis method provides higher yields of RNA and is more appropriate for lowly expressed transcripts.

Nanofluidic systems are ideal when investigating a given set of target transcripts and the use of either smaller (multi-array chip) or larger (single-array chip) number of samples allowing adaptation to the scale of the experiment. To obtain an unbiased picture of all transcripts expressed in a single worm, the obvious choice is to use RNA sequencing. If, however, the focus of the experiment is a smaller but still relatively large set of target genes, it is more cost-effective to utilize this protocol, provided the researcher has access to a nanofluidics PCR machine. The cost of the nanofluidic system reagents and a single-array chip is estimated as approximately £13 per worm, whereas the costs of the reagents for single-worm sequencing would be approximately £60 per worm, not including the sequencing costs.

When considering what PCR platform to use, the Worm-to-CT method coupled to nanofluidic qPCR offers advantages with regards to time and throughput. It is possible to obtain 9,216 RT-qPCR results in approximately 2 days of work, whereas amplification of the same number of targets using a standard qPCR platform would take approximately 5 working weeks using 96 well plate assays, running four plates a day. However, if the number of targets to be tested is smaller, then it is more cost-effective to use Worm-to-CT coupled with a standard qPCR machine. The single-array chips cannot be rerun, so running empty wells decreases cost-efficiency.

One limitation of the method is the potential formation of primer-primer dimers during the multiplexing step, but this occurs in less than 1% of the cases. Although the Worm-to-CT protocol is efficient and provides reliable results when applied to single worms, there is a failure rate of about 5%, which likely corresponds to cases where the worm remains trapped in the cap or the top of the tube during the harvesting step.

Together, this versatile and reliable method offers increased throughput and sensitivity compared to more standard techniques. This method can be very useful for validation of high-throughput screens and is an excellent choice to either monitor or validate single-worm gene expression levels. This method can be applied to other challenging techniques, such as the quantitation of gene expression from isolated tissues. For example, isolation of full tissues, such as the intestine, gonads, or cells isolated by FACs, provides enough material to perform RNA sequencing experiments. However, limited amounts of material often lead to duplicated reads, which precludes quantitation of rare transcripts. In this scenario, using nanofluidics-based technology should provide added sensitivity to the experiments and increase cost-efficiency if the researchers need to monitor only a subset of all transcripts in those tissues or cells.

## Supplementary Material

Table S1 Primers from the database of RT-qPCR primers.

## Figures and Tables

**Table 1:** Plan layout for a multi-array chip. The table above shows a simple layout that can be utilized when planning a multi-array chip run. On the left are the spaces that should be filled with the primer targets of interest and on the right are spaces that should be filled with the samples of interest. Each assay and sample array is paired number-wise through the chip.

	Primers								Samples					

Assay 1								Sample 1						
														

Assay 2								Sample 2						
														

Assasy 3								Sample 3						
														

Assay 4								Sample 4						
														

Assay 5								Sample 5						
														

Assay 6								Sample 6						
														

**Table 2:** Plan layout for a single-array chip. The table above shows a simple layout that can be utilized when planning a single-array chip run. On the left are spaces that should be filled with primer targets of interest and on the right are spaces that should be filled with the samples of interest.

Primers								Samples						
		
	1	2	3	4	5	6			1	2	3	4	5	6
A								A						
B								B						
C								C						
D								D						
E								E						
F								F						
G								G						
H								H						
I								I						
J								J						
K								K						
L								L						
M								M						
N								N						
O								O						
P								P						

**Table 3:** List of RT-qPCR primers used in this study.

Gene	Sequence Forward	Sequence Reverse	PCR efficiency (%)	R2
ama-1	GGTGGATACAGCCCATCGAG	CGAGGATGGAGTGTACGTCG	101	0.992
atg-1	CTGCCAAGACGGACTACGCT	TGCTGCCACATACGACGAAG	148	0.849
atg-12	GGACACTGTTGCTTCCTTCATCT	AAGAAGAGTGAGTTGTTGGCTTGA	97	0.923
atg-5	AAGACGAGTCGGCACAGTTGT	CGATCGTCCAGAACTCGTCA	100	0.9599
atg-7	CAGCCACGACGATAATACAACAG	CCACAAGCAACACATTGATCG	112	0.8555
atg-9	GGCTCATCCACAAGGTCACTG	TCGGCAGGTGAATCGTGATAC	115	0.901
C12C8.1	TCGATGAAGTTGTCTTGGTTGG	AGGCTACTGCTTCGTCTGGATT	110.1	0.978
C30C11.4	GAGACTCGTTCCAAGACTGATGC	CGGCTTCTTCTTATTGAGGATAGG	109.51	0.997
cct-1	ACATCTCATTCACTTCCGACTCG	CAACAACCAGCTCTCCGAAGA	107.88	0.99
cct-4	CAGCCACGACGATAATACAACAG	CAGGCGGTAGAGCAGGAAGT	114.14	0.993
cct-5	CAGGAGAACCAGAAGCGTATCAC	GACCGAGAGATGTGCGAAGAG	105	0.9944
cct-6	ACCACATCCACCGTTCTCCTT	ACTCGAAGCCTTCAGTAACAATACG	102	0.9893
cdc-42	TCCACAGACCGACGTGTTTC	AGGCACCCATTTTTCTCGGA	100.3	0.995
daf-16	GAACAGTGTCCGTGGATCGT	CGGTAGTGGCATTGGCTTGA	100	0.9934
daf-21	ATTCGCTACCAGGCACTCAC	TGGTAAGGGTCTTTTCCTCCT	109	0.994
dap-1	TGCCAGCTAAGATGGAAAGA	ACACCGCTATTAGCATCACG	112	0.9942
dnj-11	AGCAAGCCGACAAGGAGACA	CATCTCTCCACGGTTCCAGGT	107	0.9732
dnj-12	TCACTGCGACAGTTGTAATGGAG	CCAGGCGTGATTCCAACTTC	105.81	0.99
dnj-13	CGGATAAGAATAAGGAAGCTGGAG	AGTCCTTCCTCTCCGAATTGATC	103	0.98
dnj-15	TCGGTTGGATCAGGATGAGC	TCTCCTCGTCAGTCGCCATT	112.15	0.98
dnj-16	AATATGATGAAGCTGTGGCGAA	TTATACGGTTCTTGAGACTTCTGGAG	98	0.9062
dnj-19	TTCACGTTCTTCCTGGCATG	GACAACATCTCCTGGCTCACC	98	0.9763
dnj-22	AAGAAGCCGAACGTAGATTCATTG	GGTGTTCCTGAGCCATTCGA	101	0.9871
dnj-23	TAAGGCTGAGAAGGAGGCAATC	AGTGAATCGAGGAACGACGTG	102.56	0.99
dnj-24	AATGGCGATGAACATATTGTTGA	ATGTTGGCGGTGTTGTTACTGT	107.08	0.99
dnj-25	TTGACGAAGAACCTCCAGCAC	TCCATTAGACCGAACGCAGAC	102.3	0.99
dnj-26	AGTACGCAAGGCGACGACAT	AGGCTCATTGCTGGTAGAGTGTG	105.67	0.99
dnj-29	CGTTGCTTCAGCTTCCACAC	ATGGCACGAGAATACACGCTT	100.09	0.99
dnj-30	TTGCTGATCGAGAGAAGAAGAGAA	TTCTCTGCGGTCTGGTTGCT	106.71	0.99
dnj-4	CAGCGAACACATTACGAAGTGC	CGGATGAGTTGTCAGGATGGA	95.37	0.99
dnj-5A	CTCCATCTCAAGCAAGAAGTTGTAAG	CGTGTAATAGGTAGAAGTCAATCCGA	102	0.9898
dnj-5B	CTCCATCTCAAGCAAGAAGTTGTAAG	CGTGTAATAGGTAGAAGTCAATCCGA	102	0.9834
dnj-7	GCACGGATAGTACGGACACCA	CTTGAGGCATTCTCGGATAACG	110.61	0.99
dnj-8	TTCTCACAATATGTGTATCCTCTCAGAA	CAGTTGGAAGTGTATGCTCCAGTAG	100.53	0.99
dnj-9	CGCAATTCTGAATGTTCCGA	CAGCGTCCTTCTTCTCGTCAT	94.4	0.99
ERCC_171	CCATTGGATGAAGCCCAGGA	CGTAATCAACGCCGCAACTT	109	0.995
ERCC_25	GGTTCGAGTACCCAGAGCAG	AGCCGTAGACGCAACTCATT	91	0.932
ERCC_35	GGCTCTCTGATGCTACGACC	CTACCGGTTACGACGCAGTT	78	0.9834
ERCC_42	CATGGTGAGGAGCTGGTGAG	ATTGGAGGGCACTTACCTGG	103.7	0.998
ERCC_53	AACAAATCCTTTCCCTGCCTG	TTGCTCACCAGCTACTGGAAAT	100.5	0.993
ERCC_60	TGTGTTCTTGTGTGAAGTGGC	AAAAGAGGGGGAGGAGCCAA	82	0.9911
ERCC_9	GCTAGTCTCGCGCAGTACAT	AGAGCTGCGTCACATACGTC	98	0.9894
F11F1.1	GCTGTTGGTGAGGATGGAGA	TCATCATATCGCCGTCCAAT	100.8	9.979
F18C12.2	TCTTGCCAGAAGCGAGAACTC	GATTGCTCATTGGTGGTGGAT	111	0.985
F21F3.3	CCATTTCTCCGAATTCGTCT	GGGTAGAAGTTGGCCTCAAT	93.1	0.96
F44E5.4	GAATGGAAAGGTTGAGATCCTCG	GCTGCATCTCCAACCAATCTT	97.8	0.992
F48D6.4b	AAAGTGTCGAACCCTGTTCC	GCATTGTCGGTCTTGTTCTTC	94	0.99
F55H12.4	TTGGCAAAGGAACCAGCTAT	GTTTGCGAACCATACCACCT	105	0.964
gapdh	TGGAGCCGACTATGTCGTTGAG	GCAGATGGAGCAGAGATGATGAC	100.2	0.996
hsf-1	TGGTCTAACTCGAACAGAATCAGA	TCCACAGTTCTTGCCGATTG	105.59	0.99
hsp-1	CACTGTTTTCGATGCCAAACG	TCCTTCGGCAGAGATGACCT	107.76	0.988
hsp-12.3	TGGAGTTGTTAAGGTTCTGGACT	CCAATGTTCTTCACATCAATCTCGT	95.54	0.999
hsp-16.1	ATGGCTCAGATGGAACGTCA	TGGCTTGAACTGCGAGACAT	102	0.984
hsp-16.2	TCCATCTGAGTCTTCTGAGATTGTT	TGATAGCGTACGACCATCCAAA	102.2	0.998
hsp-16.41	TGGGGAGATTGTAAATGATGAATCC	TCAATTTTGAGCTCTCTTCCATCT	104.7	0.998
hsp-16.48/49	TGGATGAAATCACTGGATCTGT	GATTCGTCATTTACAATCTCTCCAA	90	0.9952
hsp-3	CATCGCCTACGGACTTGACA	AATGGTGAGCATGGATACATCG	106.63	0.98
hsp-4	TTCAACAAGACATCAAGCACTGG	GGCAGAGACTTCTTCAGGAGTGA	96	0.9873
hsp-6	GATTGGATAAGGACGCTGGAGA	CCGTTGGTGGACTTGACCTC	101	0.9916
ife-1	CAGCGTCTGGACTAAGGATTGC	GGATCACATCGAACAGTGGCTT	108.43	0.99
ire-1	TACTTGCCACCACGGAGACC	CGTTGCCATCGTCATCATTG	110.29	0.99
K07F5.16	AAGTGAAGGAGCACAACACGAAT	GGAATGAAGCCACGAATCGT	111.6	0.99
let-363	AACTGATAAGACCAAGGAACGAGT	GCGGAAGAACAAGATGAAGATG	110	0.962
lgg-2	CCGAGCATATAACCGTTGCC	GCTTGTTGTGGATGAAGTTGGA	116	0.963
nlp-29	AGGATATGGAAGAGGATATGGAGG	CTCCGTACATTCCACGTCCA	114.78	0.99
pha-4	GCACACTAGGAACCACGCAAG	CCGAACTGTAGAGGTAAGGAGACG	100	0.963
phat-3	ATTGTGCTGTAAGACGGCTGACT	CACTGTTCATTGGTAATGGTGGAG	95	0.8264
pmp-3	GTTCCCGTGTTCATCACTCAT	ACACCGTCGAGAAGCTGTAGA	109.34	0.99
sip-1	CGGGTTCAGCAAGAGATCGT	CCAAGTCGACGTCCTTTGGA	114.198	0.994
sma-10	AAGTTGCAAGTCTACCAAGCG	CCGGTCTTGGAGTTCCTGTG	81.75	
sma-3	TGGAAGCTCAACGGCATCTT	TCGTGGTGAACTTGCACTT	106.6	0.987
stc-1	AATCAGTACCGAGTGTCGTTGCT	TGTTCTTGTTGTTCCGTGGCT	96	0.9656
T02C5.1a	AACGAGCGTGAAATCAAGTG	GACTTTGCGATGAGCTGGT	111	0.971
T03F6.2	TTGTAGTGATGAATGTGATGAAGAAGG	GATGTGTTGCTTCGAGTTCTCATG	112	0.99
T10B5.5	ATCGGAATCAAGAAGGTCAACG	GAAGCCAGCATAACTGAACGC	101	0.9829
T14G8.3	TGTGCTGAATAAGGAGTCAAGAAGA	GTAGGAAGATTGAGGATGCGAAG	101.39	0.99
T21B10.7	CTGGACTTGACTCGGCTGAAC	CGTAACATCAGCAACCTCTCCTT	101	0.9772
T24H7.2	AGGTTGATGATGCTCCGAAGA	AGCAGTTAGTGCTTCAACTGTATAGCTT	110	0.984
T28F4.5/ dap-1	TGCCAGCTAAGATGGAAAGA	ACACCGCTATTAGCATCACG	108.45	0.998
ttr-45	CGACGGGCAAGGAATGTTCA	CGGAGTCCTGGCTTCAACTT	114.15	0.99
vps-34	TTCTCACTTATGTTAGACGCTGGAAT	TTGGTAGCTGCTTCATCCGA	92	0.907
Y22D7AL.5	CCAAGGACGTCAAGTTCGGA	TCACGTTTCTTCCTTTTGGGC	106	0.9929
Y45F10D.4	GTCGCTTCAAATCAGTTCAGC	GTTCTTGTCAAGTGATCCGACA	103.31	0.997
Y51F10.7	ATGCGAATTTGCACACAACT	GTACGGCTTATTAGCAGCAATC	103	0.9825
Y54E10BL.4	TTCGCTGGAGCAAGACAACA	TCTTGTGAAGCAGAATGGTCGA	106.02	0.96
Y55F3AR.3	GACACTGTGATTCTGTGGCTGTTC	CGGATTATGATTGTGGCGACTT	95	0.984
ZK637.12.2	GATGGACAACGAGAAGCTGA	GGGATTTGCATCTTCAACTG	92	0.927
